# A diverse epigenetic landscape at human exons with implication for expression

**DOI:** 10.1093/nar/gkv153

**Published:** 2015-03-12

**Authors:** Meromit Singer, Idit Kosti, Lior Pachter, Yael Mandel-Gutfreund

**Affiliations:** 1 Department of Computer Science, University of California at Berkeley, Berkeley, CA 94720 USA; 2 Faculty of Biology, Technion – Israel Institute of Technology, Haifa 32000, Israel; 3 Department of Mathematics, University of California at Berkeley, Berkeley, CA 94720, USA; 4 Department of Molecular & Cell Biology, University of California at Berkeley, Berkeley, CA 94720, USA

## Abstract

DNA methylation is an important epigenetic marker associated with gene expression regulation in eukaryotes. While promoter methylation is relatively well characterized, the role of intragenic DNA methylation remains unclear. Here, we investigated the relationship of DNA methylation at exons and flanking introns with gene expression and histone modifications generated from a human fibroblast cell-line and primary B cells. Consistent with previous work we found that intragenic methylation is positively correlated with gene expression and that exons are more highly methylated than their neighboring intronic environment. Intriguingly, in this study we identified a unique subset of hypomethylated exons that demonstrate significantly lower methylation levels than their surrounding introns. Furthermore, we observed a negative correlation between exon methylation and the density of the majority of histone modifications. Specifically, we demonstrate that hypo-methylated exons at highly expressed genes are associated with open chromatin and have a characteristic histone code comprised of significantly high levels of histone markings. Overall, our comprehensive analysis of the human exome supports the presence of regulatory hypomethylated exons in protein coding genes. In particular our results reveal a previously unrecognized diverse and complex role of the epigenetic landscape within the gene body.

## INTRODUCTION

Regulation of gene expression occurs at many different stages, such as transcription, RNA degradation, translation and protein degradation. Cytosine DNA methylation is a heritable epigenetic mark present in many eukaryotes (1–5), and is known to be associated with transcription rates through a plethora of different mechanisms (6–10). In vertebrate genomes the majority of CpG dinucleotides are methylated, with the exception of CpG islands that are mostly unmethylated (11). While DNA methylation at gene promoters is strongly associated with gene silencing (6,11,12), methylation in gene bodies has been mostly shown to associate with transcription elongation (3,8,13,14) and has been speculated to have a functional role in this context (8,9). In recent studies different correlations between transcription and intragenic DNA methylation have also been described (15,16). Overall, gene body methylation is highly abundant in human (3) and is conserved across plants and animals (1,2).

Eukaryotic genes include exons and introns that are processed by the spliceosome, which is responsible for intron excision and ligation of exons via the process of splicing (17). In metazoan, alternative splicing, the process which produces distinct mRNA and protein isoforms from single pre mRNA transcripts, is highly widespread (18). The strong coupling between transcription and splicing suggests that chromatin structure and DNA methylation may also regulate splicing and specifically alternative splicing (19–21). Consistent with the generally high GC content of exons, it has been shown that nucleosomes are preferentially positioned in exons (22–26). Analyses performed on different cell types from different organisms indicate that certain histone modifications, particularly H3K36me3, are elevated not only within genes, but specifically at exons (22–28). Furthermore, nucleosome positioning has been shown to be associated with DNA methylation patterning throughout the genome, and particularly at exons (29).

DNA methylation levels have been shown to be on average higher at exons than at flanking intron regions in human (30,31) and in a variety of other organisms (2,5,29,32), leading to speculations regarding the role of DNA methylation in splicing (9,29,31). Differential DNA methylation levels have been specifically shown to be higher for alternative exons with similar levels of GC content as their flanking introns, which are also weakly marked by nucleosomes (31). A correlation between DNA methylation and alternative splicing has also been discovered in honeybees (*Apis mellifera*) (32), where alternatively spliced genes were found to be methylated in many cases. Specifically, included exons were found to be highly methylated when compared to skipped exons. Two recent works have established a direct connection between DNA methylation and splicing efficiency in human, through the blockage of binding-sites of CTCF (33) and the recruitment of MeCP2 (34). These exciting studies have suggested a causal relationship between DNA methylation and alternative splicing. However, the major role of elevated DNA methylation levels at exons relative to their surroundings remains unclear.

In this study, we investigate the relationship between DNA methylation and gene expression in two different cell types, the IMR90 human fibroblast cell-line and uncultured CD19+ B cells. Consistent with other studies, we observe that most exons tend to be more methylated than their intronic surroundings. Intriguingly, the differences in the methylation levels are significantly higher for low expressed exons, independent of their inclusion rate. Interestingly, among all exons we observed a substantial number of hypomethylated exons being significantly less methylated than their intronic flanking regions. The methylation rates of these exons are not correlated with expression rates, suggesting the presence of a different epigenetic landscape for this subset. An analysis of 28 histone modifications across the same set of exons revealed that at high expression, hypomethylated exons are significantly enriched with a variety of histone modifications, composing a unique histone code represented by many histone modifications which have not been previously characterized within the gene body. A notable exception was H3K36me3, the hallmark of active transcription in gene bodies (35) which was significantly lower at hypomethylated exons. Further, we found that a substantial set of the hypomethylated exons within highly expressed genes overlapped DNase-I hotspots suggesting their possible role as enhancers. Overall, our results strongly support a significant but non-linear dependency between DNA methylation, histone modifications, and expression at exons in human cells.

## MATERIALS AND METHODS

### Exon datasets

Exon coordinates were determined using the RefSeq annotated set for human (hg18). Our set of exons is defined as internal exons that are not first or last in any of the transcripts of the RefSeq annotation. We restricted our analysis to the set of 34,336 exons that are flanked by introns of length greater than 600 nucleotides that do not overlap annotated transcription start sites or ends. The final set of exons was associated with 8,598 genes. We partitioned the exons into a ‘constitutive’ and ‘alternative’ set by the definition used in (22). Each exon was assigned an EST based inclusion rate by the proportion of ESTs overlapping the region that supported inclusion of the exon. Exons with EST inclusion rates of 1 were considered constitutive and exons with inclusion rates between 0.05 and 0.95 were considered alternative. Of the 34,336 exons analyzed, 31,854 were determined constitutive and 2,482 were determined alternative. Among the 31,854 constitutive exons, 418 exons overlapped transcripts in the opposite direction. All analyses were conducted on the constitutive exon set, unless stated otherwise.

### Promoter annotations

For each gene we extracted the region 1000 bp upstream to the TSS (based on the RefSeq annotation for the human genome (hg18)). In each promoter region we searched for the TATA element (TATAWAWR) following the procedure described for annotating canonical/non-canonical promoters in human and yeast (36). Among the 8,598 genes, 1,710 were annotated in this study to possess canonical promoters.

### DNA methylation scores

DNA methylation rates were computed from the publicly available datasets (3) and (37). The whole-genome bisulfite datasets for IMR90 and for B cells were downloaded and the methylation state of each CpG site with read coverage > 4 was determined as the proportion of reads covering the position that were converted at the relevant position by bisulfite. Regional DNA methylation rates were then determined by averaging of the site-specific scores.

Throughout this manuscript we considered regional DNA methylation scores (rather than site-specific rates) and applied a coupled-region correction to the comparative analysis (discarding instances in which one or more of the adjacent regions compared does not have a measurable value, due to the lack of CpG sites). We followed this approach in order to avoid biases originating from the differences in selection rates between exons and introns (see Supplementary Note 1).

### Expression and inclusion rates

Reads from the RNA-Seq datasets for the IMR90 cells (3) and the B cells (37) were separately aligned to the human transcriptome with Tophat (38), and transcript-specific expression scores (FPKMs) were assigned by Cufflinks (39), using the UCSC hg18 transcriptome as a reference. Exon-specific expression scores were assigned by summing up the FPKMs of the transcripts in which the exon was included. Since we use constitutive exons throughout the study these expression values are consistent with the gene expression. A set of high expressed exons and a set of low expressed exons were determined as the set of exons at the top and bottom 20th percentile of expression rates, respectively, and was restricted to exons that had FPKM > 0. For cassette exons the inclusion rate of each exon was defined as (exon-specific expression score)/(gene expression score), where the gene expression score is the sum of expression scores over all transcripts that overlap at some region.

We computed empirical P-values for observed Pearson correlations by randomly permuting the values in one of the two sets 10,000 times, and recording the number of times in which the Pearson correlation for a random permutation was larger or equal to the correlation observed.

### Comparison of exon methylation to flanking regions

We computed average methylation rates at the 34,336 exons (see above), along with the methylation rates of the flanking upstream and downstream 200 nucleotide intron regions. Regions that did not have any measurable value within their range (either due to no CpGs in the reference genome or due to lack of sufficient data available) were marked as ‘missing data’. In order to avoid biases in the comparative analysis we discarded regions at which either the exon or one of the flanking regions was marked as ‘missing data’ (see Supplementary Note 1). The numbers of regions that were considered in each comparative analysis following this correction are listed in Supplementary Tables S1 and S2. Boxplots were generated for the filtered sets with R http://www.r-project.org/.

To assess the significance of one set of exons (low 20% of expression/inclusion) having larger differences in methylation rates relative to their flanking introns in comparison to a second exon set (high 20% of expression/inclusion), we computed the differences in methylation rates between the exons and their surroundings in each sets, and computed the *P*-value for significance using the Wilcoxon rank sum test

### Comparison of exon-specific upstream and downstream methylation differences

To evaluate the significance of the methylation rates at exons being higher than their immediate surroundings, we assigned *P*-values in the following manner: the number of times that an exon was more methylated than both flanking intron regions was recorded (*k*), out of all instances at which all three methylation values were different from each other (*n*). The *P*-value was then computed to be the probability of observing *k* or more instances in which the exon's methylation rate is larger than that of both introns, under the null model that the probability of such an occurrence is 1/3.

Further to assess whether the magnitude of methylation differences is exon-specific, the differences between the methylation rate of the exon and that of its flanking intronic regions was computed by subtracting the methylation value of an intronic flanking region from that of the exon. Significance of the correlation rates of the differences was computed by recording the number of times the Pearson correlation of randomly permuted differences exceeded the correlation observed (across 1000 iterations).

### DNA composition and GC content analysis

The nucleotide content (A, T, G, C) was calculated for each exon and its flanking intronic regions. In addition the GC content at each region was computed as the count of G and C nucleotides divided by the total number of nucleotides within the region. The differences between each exon and its flanking intron regions were calculated and recorded. The Wilcoxon rank sum test was employed to evaluate whether the distributions of the differences differ between groups.

### Exonic distribution throughout the genes

Each exon was localized to its gene using the knownGene table from UCSC genome browser. Each gene was divided into three equal length segments: 5′ segment, middle segment and 3′ segment, excluding the first and last exon. The exons were then assigned to one of the segments according to their relative location in the gene.

### Histone modification analysis

Publicly available ChIP-Seq datasets of 28 different histone modifications for IMR90 from were downloaded from the NIH Epigenome Roadmap Project (http://www.epigenomebrowser.org/). We considered the regions 200 nucleotides upstream and 100 nucleotides downstream of the intron-exon junctions, and the regions 100 nucleotides upstream and 200 nucleotides downstream of the exon-intron junctions in the analyzed exon set (only regions with available data across the entire region were analyzed). The density of each histone modification was analyzed separately across the sets of methylated and hypomethylated exons, at different expression rates. To analyze the difference in modification intensity between the hypomethylated and methylated exon sets we computed the mean and standard deviation of the normalized mean values across all 28 modifications. The normalized mean values were computed by dividing the computed mean values of each modification (at base-pair resolution, for both the hypomethylated and methylated sets), by the highest mean value observed for that modification.

### Chromatin, DNA accessibility and IMR90 enhancer region analysis

H3 ChIP-Seq data was downloaded from (40) (GEO accession number: GSM1135044, control condition dataset). The fastq files were downloaded from the ebi website (http://www.ebi.ac.uk/ebisearch/search.ebi?query=SRX275798&submit=&db=allebi&request_from=global-masthead).

Reads were aligned with Bowtie (http://bowtie-bio.sourceforge.net/) using the default parameters and requiring unique matches. If multiple reads mapped to the same position on the ‘+’ strand or stop position on the ‘−’ strand only a single read was maintained. At each genomic location the number of overlapping reads was recorded, and then smoothed by assigning at each position the average value across an 18bp window (centered at the position).

Regions of enhanced DNA accessibility in IMR90 were obtained from the NIH Epigenome Roadmap Project, made available in Rajagopal *et al*. (41).

Predicted enhancers for IMR90 were extracted from Rajagopal *et al*. (41) using the RFECS algorithm on the 24 histone modifications available from the NIH Epigenome Roadmap Project. Enhancer regions were defined using a window of −0.5 to +0.5 kb.

## RESULTS AND DISCUSSION

In order to investigate the relation between DNA methylation and gene expression we studied the methylation at exons and their intronic surroundings. We chose to center on the IMR90 Human fetal lung fibroblast cell-line due to extensive mapping of its epigenetic and expression landscape (3,14,42). While cultured cell-lines are an extraordinary resource for epigenetic studies, due to the relative ease of replicating experimental settings and of attaining samples, cultured cell-lines differ from non-cultured cells in their epigenetic landscape and behavior (43). Therefore, to test the generality of our findings we repeated the analysis on peripheral CD19+ B cells (37) (see Supplementary Note 2 and Table S2).

**Figure 1.**
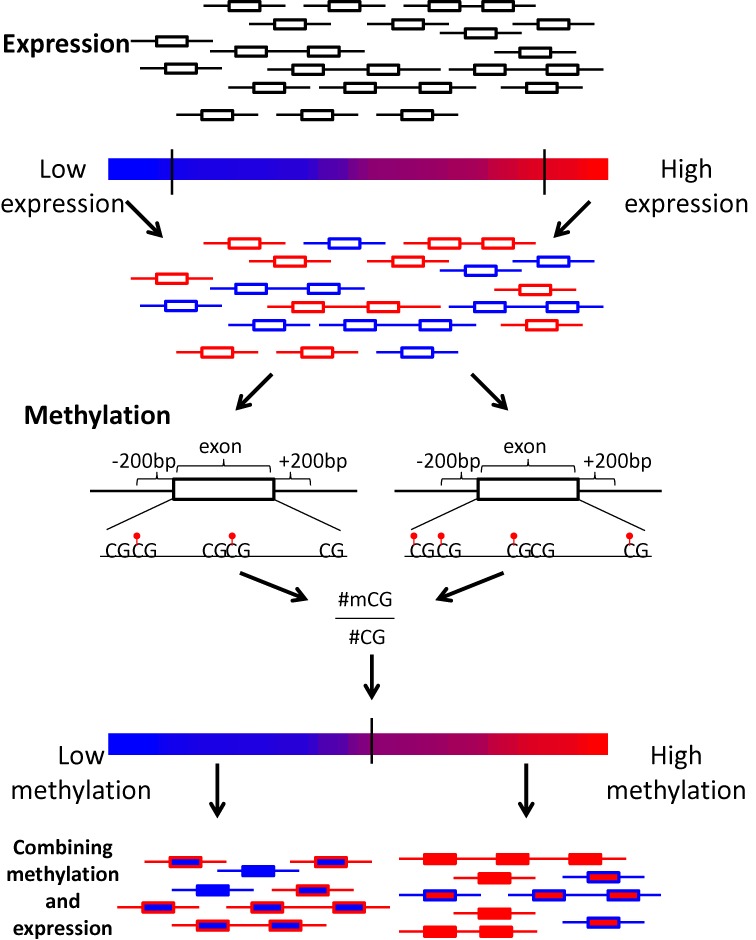
Graphical summary of the exon methylation and expression analyses. Exons are represented as rectangles. The color of the rectangle frame represents the exon's expression rate, red and blue for high and low expression, respectively. The color of the rectangle represents the methylation level, red and blue for methylated and hypomethylated exons, respectively.

We computed DNA methylation rates and expression rates for 31,854 constitutive internal exons, excluding exons that are first or last in any annotated RefSeq transcript (Figure 1, see Materials and Methods section). As previously reported for intragenic regions (3,10,14,16) the exon methylation rates were positively correlated with the gene expression rates (Pearson *r* = 0.37 for correlation of methylation rates with log of FPKM expression rate, empirical *P*-value < 0.0001).

### The difference in methylation levels between exons and their flanking introns is highly significant at low expressed genes

**Figure 2.**
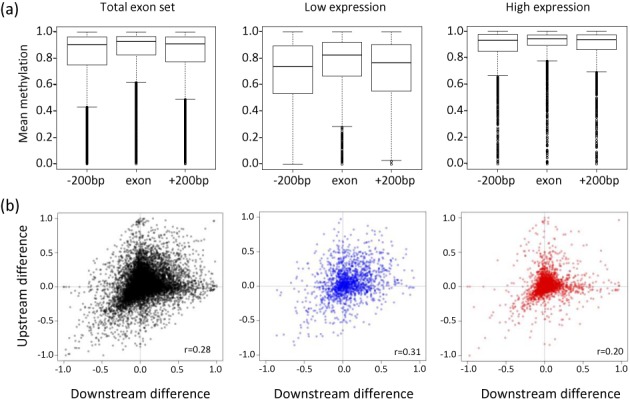
The difference in methylation between exons and introns is exon-specific. (**a**) Distribution of methylation rates at exons and their flanking upstream and downstream intron regions shown for all exons (left) the bottom 20th percentile of expression (middle) and the top 20th percentile of expression (right). The thick black lines mark medians, and the surrounding rectangles mark the range of the first and third quartiles. (**b**) The differences of the methylation rates between the exons and their upstream and downstream flanking intron regions are plotted for all considered exons (left), the bottom 20-th percentile of expression (middle), and the top 20-h percentile of expression (right).

We compared the methylation rates at exons to the methylation rates at the upstream and downstream flanking intronic regions (200 nucleotides upstream the 3′ splice site and 200 nucleotides downstream the 5′ splice site). As expected from previous studies (3,14,16,44) and from the positive correlation aforementioned, we can clearly see that overall the highly expressed exons are highly methylated relative to the low expressed exons (Figure 2a). Furthermore, in accordance with previous studies (3,30,31), we found that exons tend to be more methylated than the surrounding intron regions (Figure 2a, *P*-value = 5.2e^−19^, see Materials and Methods section). When considering independently the subsets of high expressed (top 20th percentile) and low expressed exons (bottom 20th percentile), the methylation levels of both exons and introns in the high expression set were significantly higher than in the low expression set (Figure 2a, *P*-value < 2.2e^−16^, Wilcoxon rank sum test, for the exon regions and the upstream and downstream flanking regions). By quintile measurements with a 90% cutoff on the sets of low and high expressed exons we observed that 38% of low expressed exons have lower methylation than 90% of the high expressed exons, and 36% of the high expressed exons have higher methylation than 90% of the low expressed exons.

Interestingly, we observed that the elevation in methylation at exons relative to the upstream and downstream introns was much larger in the low expressed genes (Figure 2a, middle panel, *P*-value = 3.7e^−14^, see Materials and Methods section and Supplementary Table S1) compared to the high expressed set (Figure 2a, rightmost panel, *P*-value = 0.34, see Materials and Methods section and Supplementary Table S1). A similar trend was observed for the B cells datasets (Supplementary Figure S1 and Supplementary Table S2). This significant increase in the methylation delta in low expressed relative to the high expressed exons could not be explained by the overall decrease in the methylation of the gene body *per se*. To test whether these differences between the high and low expressed exons could be related to differences in the genome content, we calculated the differences in GC content for the exons and the flanking intron regions in each exon set independently. As expected, the GC content of the exons was significantly higher than that of the introns. Nevertheless, while overall the highly expressed exons had higher GC content, the differences in GC content between exons and introns were consistent and significant (*P*-value = 2e^−4^, and *P*-value = 1.2e^−15^ for the upstream intron–exon and exon-downstream intron, Wilcoxon rank sum test) for both intron-exon differences in the high and low expressed exons (Supplementary Tables S1 and S2). These results are in line with earlier genomic studies demonstrating that highly expressed genomic regions tend to have elevated GC content (37,45). Overall, these observations suggest that elevated DNA methylation at exons are not dependent on the transcription rate and rather may be due to the different sequence characteristics between exons and introns, leading to different levels of basal methylation. These results are consistent with previous work demonstrating that other epigenetic marks such as nucleosomes are present in exons independently of the transcription rates and splicing activity (29).

To further examine whether the differences observed between the methylation level of the low expressed exons and their neighboring introns are restricted to the exon/intron boundaries, we expanded our analysis to mid-intron regions (see Materials and Methods section). We observed that the methylation rates at mid-intron regions were either similar or lower than the methylation rates of the corresponding intron regions immediately flanking the exon/intron boundaries (Supplementary Figure S2). These results indicate that the differences we observed are an inherent feature of exons.

To confirm that the methylation differences observed between the exons and their surroundings are consistent for individual exons we computed the correlation of the difference in methylation between an exon and its upstream flanking intron (upstream difference) with the difference in methylation between an exon and its downstream flanking intron (downstream difference). We found that the difference in methylation between an exon and each of its flanking regions is positively correlated (*r* = 0.28, *P*-value < 0.001), indicating that the extent to which exons are more or less methylated than their close surroundings is a local phenomenon (Figure 2b). Notably, as demonstrated in Figure 2b, the correlation between the upstream and downstream differences in DNA methylation was more pronounced for the low-expressed set (*r* = 0.31, *P*-value < 0.001) than for the high-expressed set (*r* = 0.2, *P*-value < 0.001). This trend was also present in B cells (Supplementary Figure S3). We observed a similar behavior for the subset of exons overlapping transcripts from the opposite strand (Supplementary Figure S4) and for the subset of exons derived from genes with canonical promoters (Supplementary Figure S5).

To determine whether this observed trend is characteristic of the genes’ overall expression level or of the inclusion rate of the specific exons, we analyzed methylation levels of cassette exons ((22), see Materials and Methods section), restricting our analysis to highly expressed genes in order to get accurate inclusion rate estimates (Supplementary Figure S6). We observed that the difference in methylation between the exon and flanking intron regions at the 3′ and 5′ splice sites does not depend on the inclusion rate of the exon (*P-value* = 0.15, see Materials and Methods section). Very similar results were obtained in B cells (Supplementary Figure S7). Expanding our analysis to the mid-intron regions reinforced that this is not a local phenomenon (Supplementary Figure S8). Furthermore, we verified that the set of constitutive exons (22) partitioned by gene expression rates (see Materials and Methods section) displays the same trend as observed in Figure 2 (Supplementary Figure S9). Overall, these observations show that the significant difference in the methylation patterns between exons and introns does not relate to differences in inclusion rates.

### Hypomethylated exons are less methylated than their surroundings

**Figure 3.**
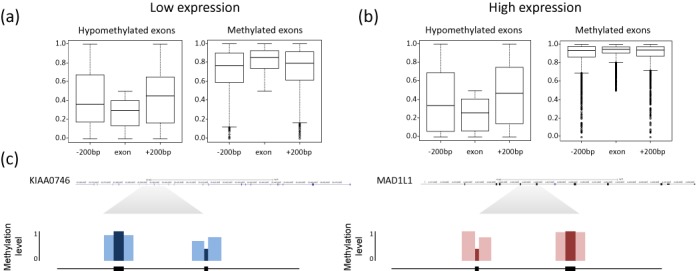
Hypomethylated exons are less methylated than their surroundings in high and low expressed genes. The distribution of DNA methylation rates is shown for exons and their flanking introns regions for (**a**) low expressed genes and (**b**) high expressed genes. In each category the distribution of DNA methylation rates is shown for hypomethylated (left) and methylated (right) exons. (**c**) Examples of the methylation rates at exons and their flanking regions are shown for KIAA0746 and MAD1L1, a low expressed and a high expressed gene, respectively, possessing a hypomethylated exons adjacent to a methylated exon. The average methylation rates at the exons and their 200 bp flanking regions are indicated.

While unmethylated CpG regions (CpG islands) are widely spread in gene promoters and have a well-established role in promoting transcription initiation, the role of hypomethylated CpG regions within the gene body is still an enigma. Based on the recent whole genome studies of DNA methylation it is becoming clear that differential methylation within the gene body has a role in multiple gene regulation processes (16). Numerous hypomethylated intragenic regions have been previously shown to be associated with enhancers (14,46) and internal promoters (10) or enhancer transcribed RNAs (eRNAs) (47). To further investigate the role of exon methylation, we divided the set of constitutive exons into two independent sets of methylated exons (13 506 exons) and hypomethylated exons (947 exons) using the threshold of 0.5 to define the average methylated state of the regions (Figure 1). The latter analysis revealed two distinct types of behaviors (Figure 3a). The set of methylated exons showed a higher methylation tendency than the surrounding introns forming a ‘hill’-like pattern, similar to the overall trend observed in other studies (31,48). On the other hand, the hypomethylated set showed lower methylation levels compared to the surrounding introns, forming a ‘dip’-like pattern. An example of hypo- and methylated exons within the same gene is shown under the box plot. The pattern observed for the latter subset (found also in B cells, Supplementary Figure S10) shows that the underlying characteristics of the majority of exons being methylated at a higher extent than their flanking regions are not applicable to the unique subset of hypomethylated exons. Notably, we found that the exon sets showing the ‘dip’ and ‘hill’ like patterns had very similar differences in GC content between the exons and the upstream and downstream introns (*P*-value = 0.06, 0.7215, Wilcoxon rank sum test, for the differences between the exons and upstream and downstream introns, respectively). We further investigated whether partitioning the exon set by various genomic characteristics recapitulates the ‘hill’ and ‘dip’ behavior. We found that partitioning by overlap with CpG islands (Supplementary Figure S11) or by GC content (Supplementary Figure S12) does not result in a ‘dip’ signature. These findings reinforce the notion that the partitioning to a ‘hill’ and ‘dip’ pattern is not accounted for by straightforward genomic characteristics.

While the methylated exon set displays smaller differences between exons and their surroundings at high expression than at low expression (Figure 3) (*P*-value < 2.2e^−16^, Wilcoxon rank sum test), in accordance with the general trend shown in Figure 2a, the hypomethylated exon set did not show such a trend (Figure 3) (*P*-value = 0.46, Wilcoxon rank sum test). Examples of hypomethylated and methylated exons along with their flanking introns for a highly and low expressed gene are shown in Figure 3c. Moreover, when comparing the expression rates of the methylated and hypomethylated exons we found that the hypomethylated exons display significantly lower expression rates than the methylated exon set (82% and 49% of the methylated and hypomethylated exons are in the top 20th percentile of expression, respectively. See also Supplementary Table S1). Overall, we show that the human genome contains a substantial set of hypomethylated exons that are distinguished from the majority of coding exons.

### Genomic characteristics of human hypomethylated exons

**Figure 4.**
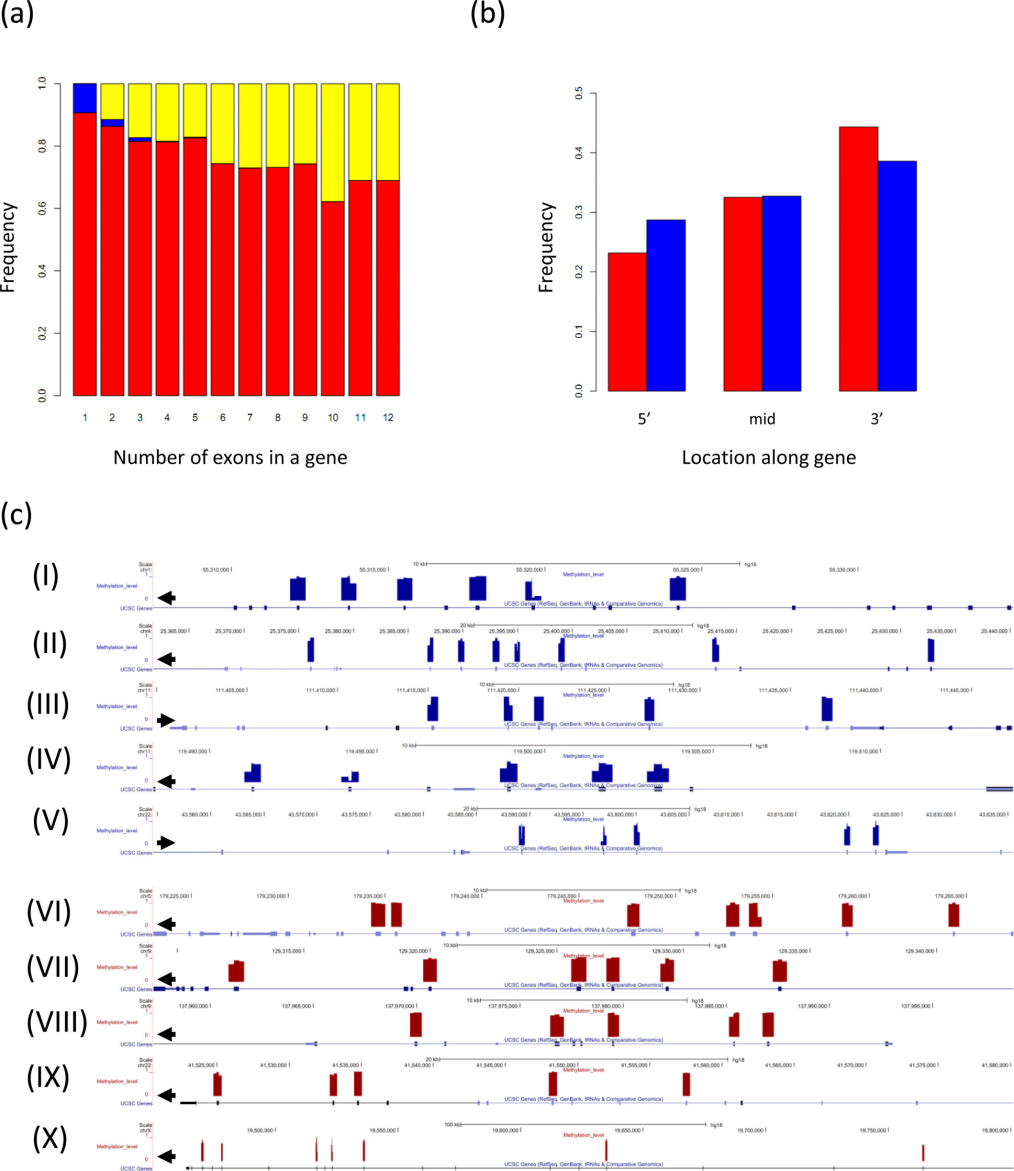
The distribution of the methylated and hypomethylated exons across the intragenic regions. (**a**) Frequencies of genes at which all analyzed exons are methylated (red), hypomethylated (blue), or at which there was a mixture of methylated and hypomethylated exons (yellow). (**b**) Frequencies of the methylated (red) and hypomethylated (blue) exons across genes. (**c**) Distribution of differentially methylated exons across the transcript in 10 selected examples of low (I–V) and highly (VI–X) expressed genes.

To gain further insight on the characteristics of the hypomethylated exon set identified in this study we analyzed the exons’ distribution across the entire genome and found that the hypomethylated exons are distributed throughout all chromosomes (Supplementary Figure S13). Overall, hypomethylated exons were found in 16.4% of the genes (1404 genes) analyzed in our study, and the number of genes including at least one hypomethylated exon is consistent with what is randomly expected given the number of exons analyzed per gene (Figure 4a, Supplementary Figure S14). Furthermore, when analyzing the location of the hypomethylated exons within the gene body, while we did notice a tendency for hypomethylated exons to be located at the 5′ end of the gene (Figure 4b and Supplementary Figure S14c for B cells) we did not detect a significant enrichment at any of the relative locations within a gene (*P*-value = 0.163, Chi-square test). Figure 4c demonstrates the distribution of differentially methylated exons throughout the transcript in 10 examples of high and low expressed genes.

Further we analyzed the length and sequence composition of all exons studied and their surroundings, looking separately at the high and low expressed groups. As demonstrated in Supplementary Figure S15, within the low expressed group the hypomethylated exons tend to be slightly shorter than the methylated exons (*P*-value = 0.007) whereas the downstream introns were on average longer (*P*-value = 0.02). Nevertheless, while consistence with previous observations the group of high expressed exons had higher frequency of G and C relative to the low expressed group (45), the methylated and hypomethylated exon sets showed very similar nucleotide composition (Supplementary Figure S16).

To test the possibility that hypomethylated exons have a role in transcriptional regulation we characterized the extent to which they overlap with DNase-I hotspots. We found that 37.6% of the hypomethylated exons in high expressed genes overlapped DNase-I hotspot regions of IMR90, i.e. were associated with open chromatin, whereas only 1% of the methylated exons within the same expression group were found to overlap with open chromatin regions (Supplementary Figure S17a). Moreover, among the high expressed exons overlapped by DNase-I hotspot regions the hypomethylated set had significantly higher score of enrichment for DNase-I signal compared to the methylated exons (median of 20.5 and 12 for the hypomethylated and methylated exons, respectively, *P*-value = 0.008, Wilcoxon rank sum test). As expected the large majority of exons in the low expressed group did not fall in open chromatin regions (5.9% and 0.4% of hypomethylated and methylated low expressed exons overlapped with the DNase-I hotspots, respectively).

### Exons are characterized by differential histone modification signatures associated with their methylation levels

**Figure 5.**
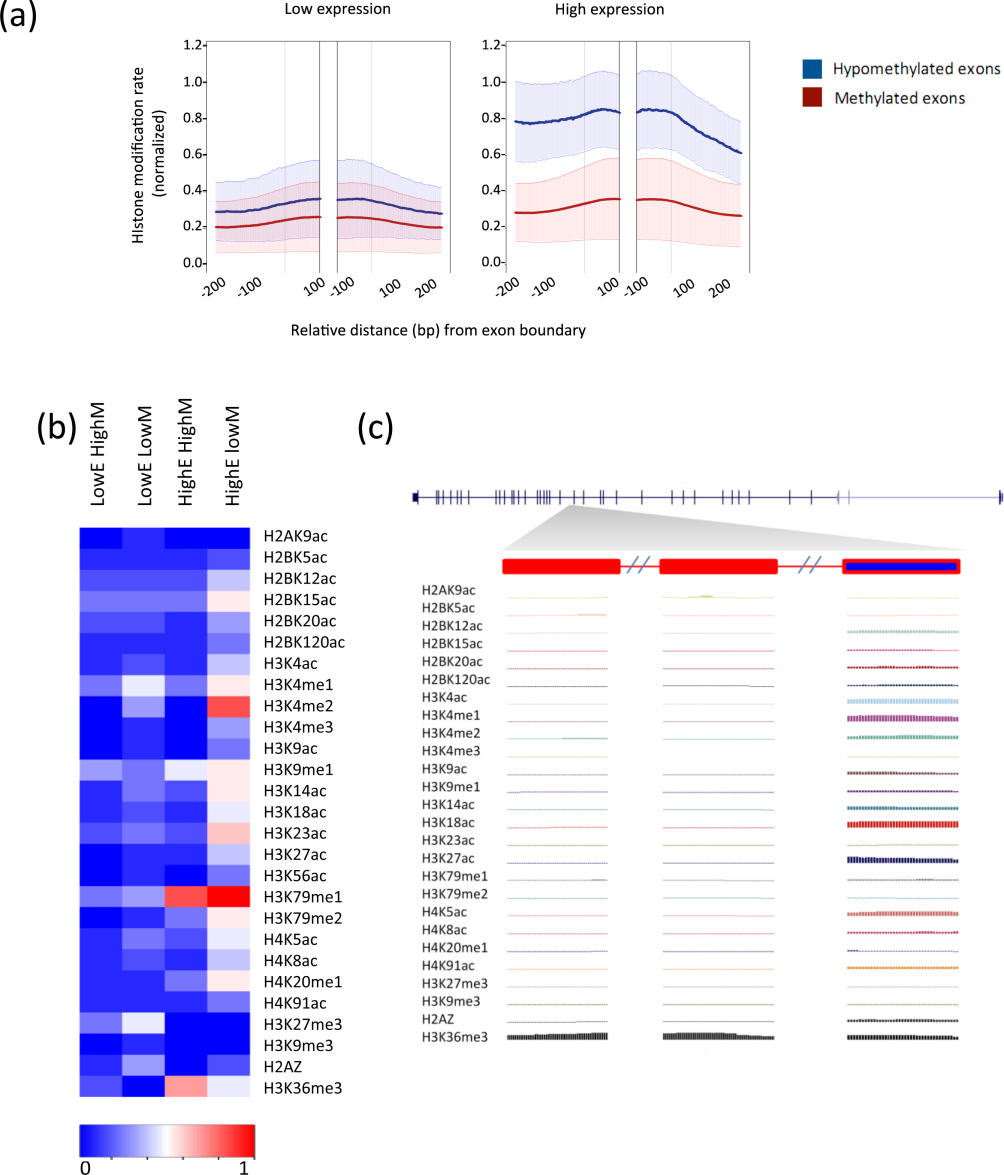
Expressed and hypomethylated exons are significantly enriched with histone modifications. (**a**) Normalized histone modification densities at the bottom 20th percentile of expression (left) and the top 20-th percentile of expression (right). Bold lines mark averages across the 28 measured histone modifications for the hypomethylated (blue) and methylated (red) exons sets. Shaded regions mark one standard deviation. (**b**) A heatmap summarizing the average density for each of the analyzed histone modifications (rows) in the four different sets (columns): methylated exons in low expressed genes, (LowE HighM), hypomethylated exons in low expressed genes (LowE LowM), methylated exons in highly expressed genes (HighE HighM) and hypomethylated exons in highly expressed genes (HighE LowM). (**c**) Density of the different histone modifications at three exons of the highly expressed *LAMA4* gene. The left and middle exons are methylated and the right exon is hypomethylated.

Following the discovery of distinguished methylation patterns at human exons and their tendency to overlap with open chromatin hotspots, we investigated further the possibility that these findings are part of a broader scope for epigenetic signatures. We computed individual histone modification rates across the boundaries of our analyzed exons sets (from the low and high expressed genes) for the 28 modifications available for IMR90 in the NIH Roadmap Epigenome Project (42), as well as H3 peak density rates (40). An analysis of all available histone modifications revealed an interesting relationship between the DNA methylation levels and the relative abundance of the vast majority of the modifications. Remarkably, at high expression rates the average normalized density of the histone marks in the set of hypomethylated exons was significantly higher than the density at the methylated exon set, revealing a strong negative correlation between the methylation level of the exon and the density of signal for the vast majority of measured histone marks (Figure 5a). This negative correlation was observed for all but three of the measured histone marks: H3K36me3, H3K27me3 and H3K9me3 (Figure 5b, Supplementary Figure S18). In addition, H3K27me3 and H3K9me3 signals were higher at low expressed exons, as expected from their role as marks of gene silencing (49) (Supplementary Figure S18o and Figure S18t). As expected H3K36me3 was enriched at exons within the high expressed genes (25). Interestingly, H3K36me3 is the only histone modification for which the mean modification densities were higher for the methylated exon set compared to the hypomethylated set at high expression (Supplementary Figure S18u). This is consistent with its suggested role in recruiting DNMTs to facilitate the methylation of intragenic DNA (35).

The difference between the epigenetic signature of the methylated and hypomethylated exon is exemplified in Figure 5c, depicting the density of 26 chromatin modifications across three exons (exons 15–17) of a randomly selected highly expressed gene, *LAMA4*, located on chromosome 6. As clearly observed, the density of the vast majority of histone modifications is significantly higher at the hypomethylated exon (exon 17) relative to its upstream methylated exon neighbors (exons 15 and 16). Here again, the only exceptions were H3K36me3 which displayed an opposite trend (i.e. higher density in the methylated exons) and the known repressive marks H3K9me3 and H3K27me3 that were found at low densities in both the hypomethylated and methylated exons in this gene.

To assure that the differences observed in histone modification density are not a consequence of the overall histone occupancy, we evaluated H3 density rates across the analyzed exon sets (see Materials and Methods section, Supplementary Figure S19). At both the hypomethylated and methylated exons we observed a subtle increase in histone occupancy at elevated expression rates compared to low expression, as previously reported by others (23). However, there was no detectable difference in the rate of histone occupancy between the hypomethylated and methylated exon sets, verifying that the observed increase in histone modification rates at highly expressed hypomethylated exons is not accounted for by an increase in the presence or detection of histone occupancy at those sets. To validate that the subtle preference of the hypomethylated exon set to the 5′ end of genes does not bias our analysis, we conducted an analysis restricted to exons from our initial exon set that were located at least 2 kb away from any transcription start site. Analysis of the histone modification rates at this subset resulted in a similar enrichment profile to that observed for the complete set (Supplementary Figure S20).

The annotation of the complete epigenetic histone modification signature at the different sets of exons showed that each is characterized by a prevalent combinatorial pattern (Figure 5b). The modifications for which the strongest relative changes in signal intensity are observed are H3K4me2 and H3K79me1.

Our results show that H3K79me1 is highly associated with both of the high expression exon sets (methylated and hypomethylated), with a slightly higher density of H3K79me1 present at the hypomethylated set (Figure 5b, Supplementary Figure S18w). This observation is in concordance with recent observations showing the role of H3K79 methylation in active transcription (50). H3K79me1 has also been annotated as enriched downstream to the TSS, specifically at bivalent promoters (51). While exons overlapping antisense transcripts were excluded from the analysis, possibly a subset of the exons enriched with the latter marks overlap unannotated bivalent promoters. The enrichment of the hypomethylated high expression exon set for a variety of different histone modifications is highlighted in the detailed per-exon heatmaps shown in Supplementary Figure S21. Looking at individual exons one can clearly see that they are composed of different histone signatures. For example, at the exons within highly expressed genes, both H3K4me1 and H3K4me3 are enriched at the hypomethylated exon set but are present at different exons within that set (Supplementary Figure S21). Interestingly, H3K4me2 is present at high density in many exons and spans both the H3K4me1 containing subset and the H3K4me3 containing subset.

Given the enrichment of a variety of histone marks at hypomethylated exons within highly expressed genes and their enrichment for DNase-I hotspot regions, a possible conjecture is that the hypomethylated exons are associated with enhancers. While the hypomethylated exons did not overlap the annotated tissue-specific human enhancers (52) we found a significant overlap between the subset of highly expressed hypomethylated exons and predicted enhancer regions in IMR90 (41). Specifically, 28.2% of the hypomethylated exons in high expressed genes overlapped with predicted enhancer regions while there was only 4.3%, 5.0% and 3.7% overlap for the methylated high expressed, hypomethylated low expressed and methylated low expressed exons, respectively. Overall, our findings define a novel set of exons within highly expressed genes that are hypomethylated, tend to be located in open chromatin region and possess a unique histone modification signature, possibly acting as internal enhancers.

## CONCLUSIONS

In this study, we investigated the relationship between DNA methylation, histone modifications and gene expression in the IMR90 human fibroblast cell-line and in uncultured CD19+ B cells. When splitting the complete exon set based on methylation level we noticed that while the methylated exons had the expected increased methylation level relative to their flanking introns, hypomethylated exons tended to be substantially less methylated than their intronic flanking regions at all levels of expression. Interestingly, in the methylated exon set the differences observed in the methylation levels between the exon and the flanking introns were significantly higher for low expressed exons and were independent of the inclusion rate of the exon.

Further analysis of histone modifications and DNase-I hypersensitivity data across the hypomethylated and methylated exons revealed that the majority of the hypomethylated exons within highly expressed genes are located in open chromatin hotspots and show extensive marking by different histone modifications independent of overall nucleosome occupancy. Moreover we detected a substantial overlap between the highly expressed hypomethylated exons and predicted enhancers that strongly implies a general functional role for hypomethylated exons in transcription regulation.

Taken together, our observations support the presence of regulatory mechanisms at hypomethylated exons, which do not relate to previously identified internal enhancers. The regulatory role of these exons could involve either an active epigenetic mechanism such as demethylation, or the rejection of the methylation maintaining machinery in those regions, both of which are mediated by extensive histone modifications. Overall, our results highlight the diverse and complex role of the epigenetic landscape of exonic regions within the gene body.

## SUPPLEMENTARY DATA

Supplementary Data are available at NAR Online.

SUPPLEMENTARY DATA
