# Supplementary material for: A diverse epigenetic landscape at human exons with implication for expression
Source: Nucleic Acids Res. 2015 Mar 12;43(7):3498–508. doi: 10.1093/nar/gkv153 (PMC4402514; doi:10.1093/nar/gkv153)
Supplement: SUPPLEMENTARY DATA [file supp_43_7_3498__index.html]

A diverse epigenetic landscape at human exons with implication for expression — A diverse epigenetic landscape at human exons with implication for expression — SUPPLEMENTARY DATA 

# A diverse epigenetic landscape at human exons with implication for expression

## SUPPLEMENTARY DATA

**Files in this Data Supplement:**

- SUPPLEMENTARY DATA
- SUPPLEMENTARY DATA
