# Supplementary material for: A diverse epigenetic landscape at human exons with implication for expression
Source: Nucleic Acids Res. 2015 Mar 12;43(7):3498–508. doi: 10.1093/nar/gkv153 (PMC4402514; doi:10.1093/nar/gkv153)
Supplement: SUPPLEMENTARY DATA [file supp_gkv153_nar-02745-a-2014-File007.docx]

**Figure S1:** Distributions of methylation rates of B cells at intragenic exons and their upstream and downstream flanking 200 nucleotides. Expression was also assessed for B Cells. (a) All exons considered (p=3.2e-36). (b) The bottom 20-th percentile for expression (p=1.1e-05). (c) The top 20-th percentile for expression (p=6.4e-06). The thick black lines mark medians, and the surrounding rectangles mark the range of the first and third quartiles

**Figure S2:** Distributions of methylation rates at intragenic exons and their upstream and downstream flanking 200 nucleotides and mid-intron regions. (a) All exons considered. (b) The bottom 20-th percentile for expression. (c) The top 20-th percentile for expression. The thick black lines mark medians, and the surrounding rectangles mark the range of the first and third quartiles

**Figure S3:** The differences of the methylation rates between the exons and their upstream and downstream flanking intron regions are plotted for the B cells for (a) all exons, (b) low-expressed exons, and (c) highly expressed exons. The distribution of the differences (both upstream and downstream) had a larger variance in the lower expressed exon set (0.04) compared with the highly expressed set (0.03).

**Figure S4:** Distributions of methylation rates of a subset of exons overlapping transcripts from the opposite strand and their upstream and downstream flanking 200 nucleotides. (a) All exons considered. (b) The bottom 20-th percentile for expression. (c) The top 20-th percentile for expression. The thick black lines mark medians, and the surrounding rectangles mark the range of the first and third quartiles.


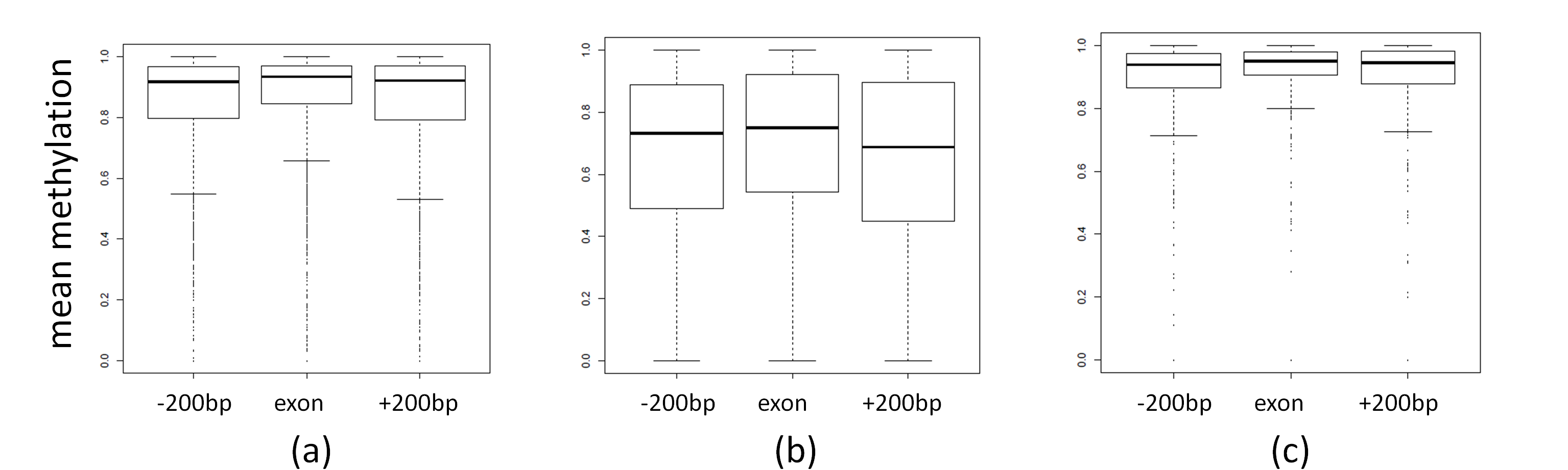


**Figure S5:** Distributions of methylation rates of a subset of exons from genes under canonical promoters and their upstream and downstream flanking 200 nucleotides. (a) All exons considered. (b) The bottom 20-th percentile for expression. (c) The top 20-th percentile for expression. The thick black lines mark medians, and the surrounding rectangles mark the range of the first and third quartiles.


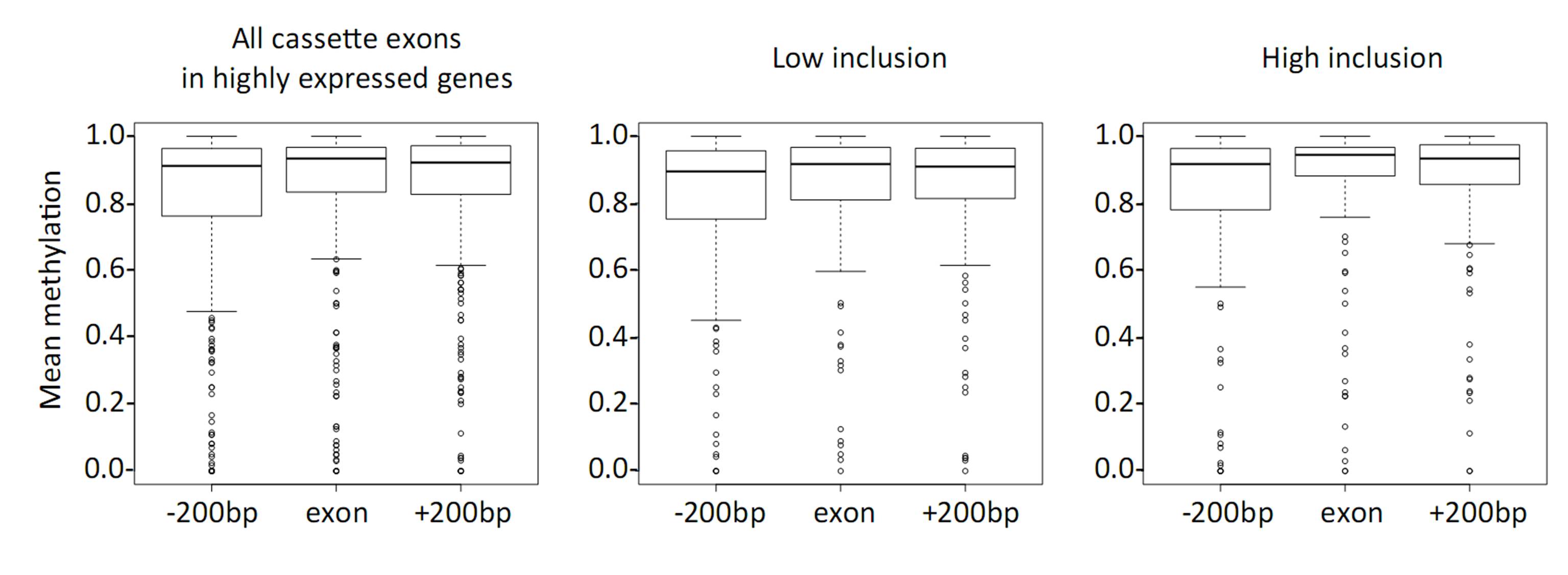


**Figure S6:** The difference between the methylation rate of cassette exons and their flanking introns does not depend on the exon inclusion rate. Distributions of methylation rates at intragenic cassette exons of highly expressed genes shown for all cassette exons at highly expressed genes (left), and for the subsets of bottom 20-th percentile of inclusion rates (middle), and top 20-th percentile of inclusion rates (right). The analysis is restricted to highly expressed genes (top 20%) because of possible inaccuracies in inclusion rate estimates for lowly expressed genes. The thick black lines mark medians, and the surrounding rectangles mark the range of the first and third quartiles.

**Figure S7:** Distributions of methylation rates at intragenic cassette exons of highly expressed genes in B cells. **(a)** All cassette exons considered. **(b)** Bottom 20-th percentile of inclusion rates. **(c)** Top 20-th percentile of inclusion rates. The difference in methylation between the exon and flanking intron regions at the 3’ and 5’ splice sites does not depend on the inclusion rate of the exon (p=0.45, see methods). The thick black lines mark medians, and the surrounding rectangles mark the range of the first and third quartiles

**Figure S8:** Distribution of methylation rates at intragenic cassette exons and their upstream and downstream flanking 200 nucleotides and mid-intron regions. (a) all exons considered, (b) bottom 20-th percentile of inclusion rates, and (c) top 20-th percentile of inclusion rates. The thick black lines mark medians, and the surrounding rectangles mark the range of the first and third quartiles

**Figure S9:** Analysis of constitutive exons by genic expression rates. Distribution of methylation rates at intragenic constitutive exons and their upstream (left) and downstream (right) flanking 200 nucleotides and mid-intron regions. (a) all exons considered, (b) low expressed, and (c) high expressed exons by gene expression rates. The thick black lines mark medians, and the surrounding rectangles mark the range of the first and third quartiles

**Figure S10:** Distributions of B cells methylation rates at intragenic exons and their flanking intron regions for methylated (left) and hypomethylated (right) exons. (a) All exons considered. (b) Bottom 20-th percentile for expression. (c) Top 20-th percentile of expression. The thick black lines mark medians, and the surrounding rectangles mark the range of the first and third quartiles

**Figure S11:** Partitioning of intragenic exons by overlap with CpG islands. (A) 31,375 exons not overlapping CpG islands (14,079 after normalization) (B) 479 exons overlapping CpG islands (408 after normalization). The thick black lines mark medians, and the surrounding rectangles mark the range of the first and third quartiles

**Figure S12:** Partitioning of intragenic exons by GC thresholds. The thick black lines mark medians, and the surrounding rectangles mark the range of the first and third quartiles.


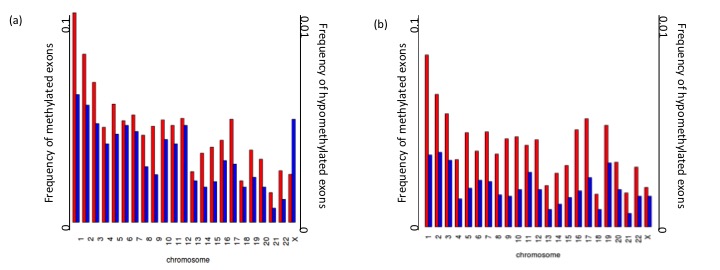


**Figure S13:** Distribution of methylated and hypomethylated exons (in red and blue accordingly) across chromosomes in (a) IMR90 cell line (b) B cells.


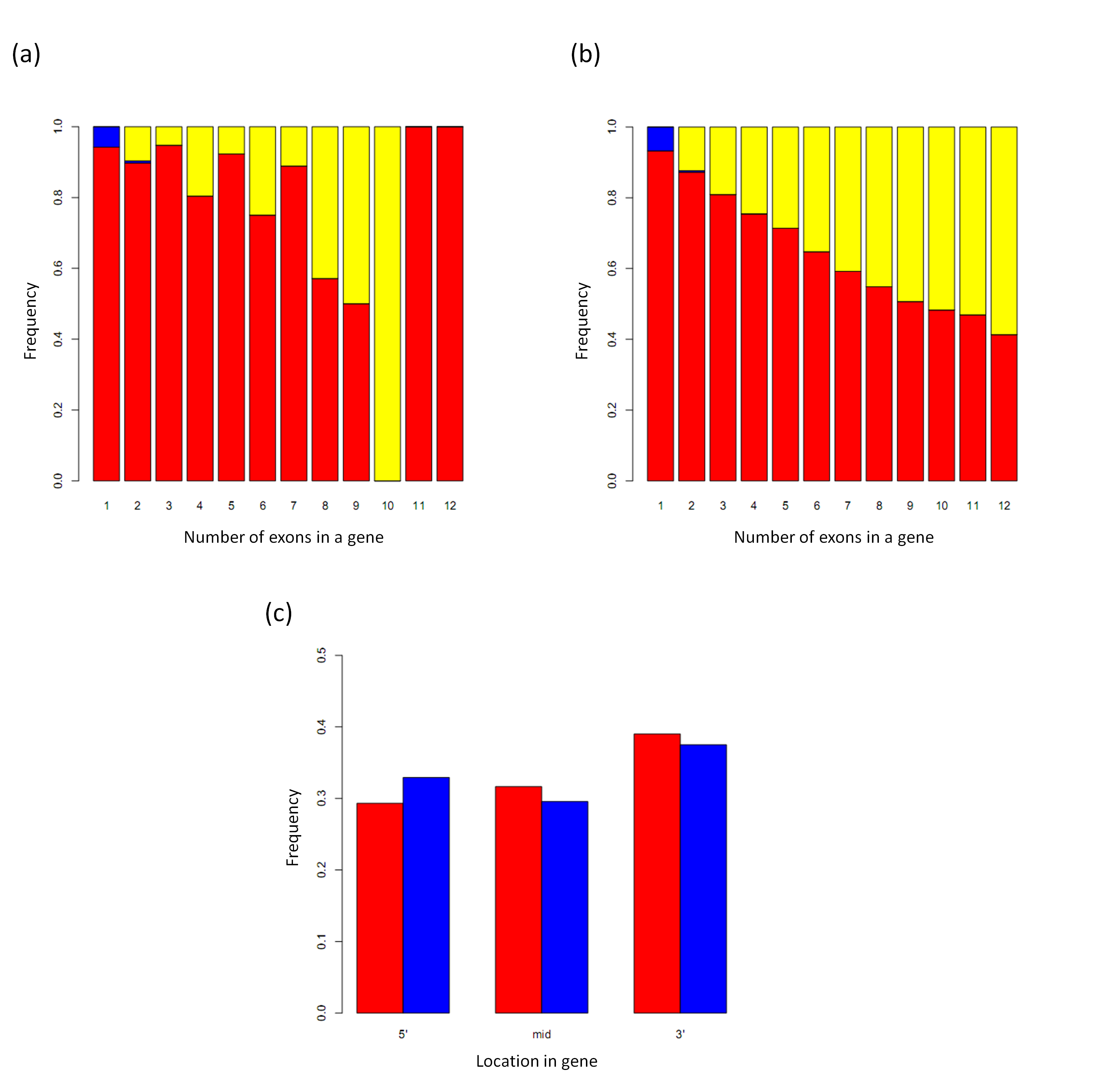


**Figure S14:** (a) Frequencies of genes in B cells at which all analyzed exons are methylated (red), hypomethylated (blue), or possessing at least one methylated and one hypomethylated exons (yellow). (b) random distribution. Genes with methylated exons are in red, genes with hypomethylated exons only are in blue and genes with methylated and hypomethylated exons are in yellow. (c) Location of methylated and hypomethylated exons (in red and blue accordingly) along the gene in B cells.


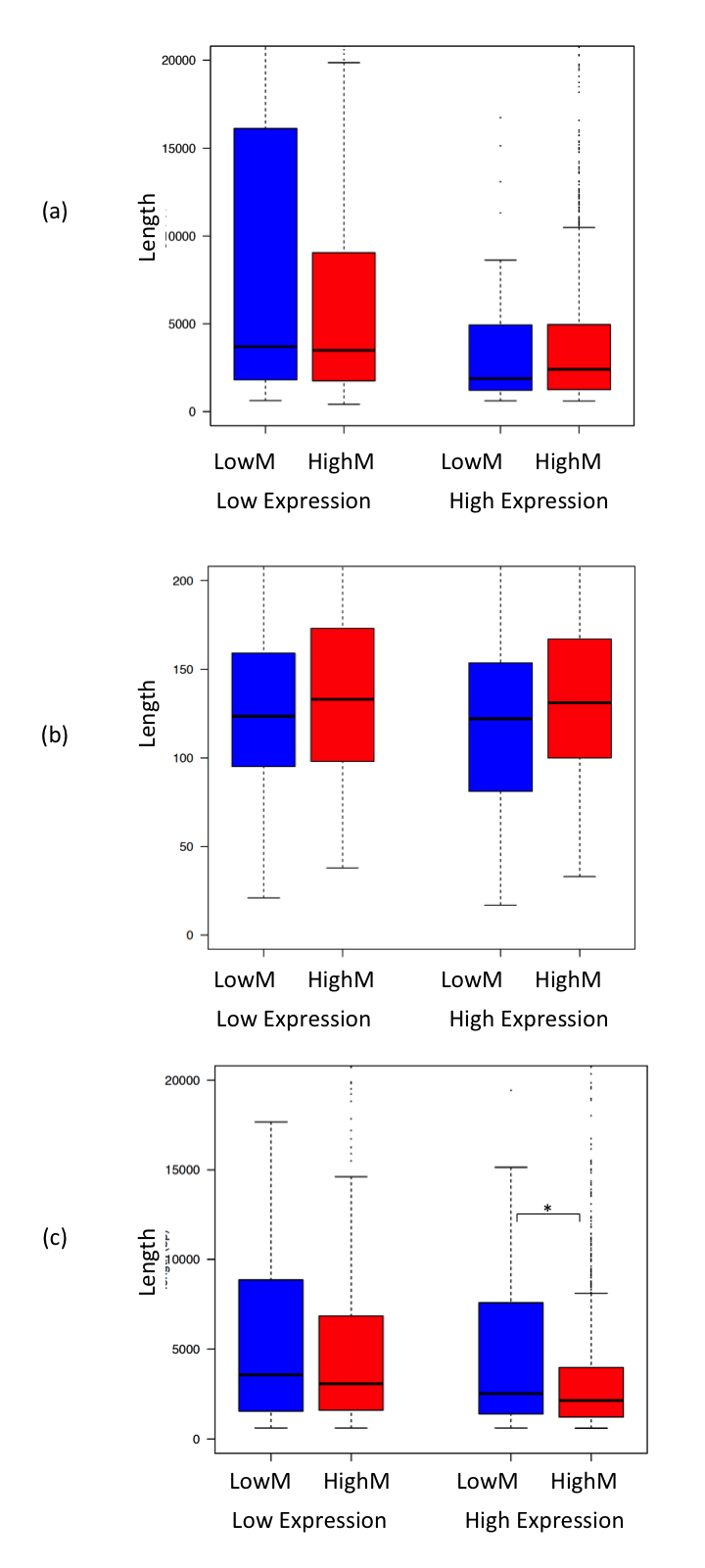


**Figure S15:** Length distribution of exons and their surrounding intron. (a) upstream intron, (b) exon and (c) downstream intron for bottom 20th percentile for expression (left) and top 20-th percentile for expression (right). Methylated and hypomethylated exons in each plot are marked in red and blue, respectively.


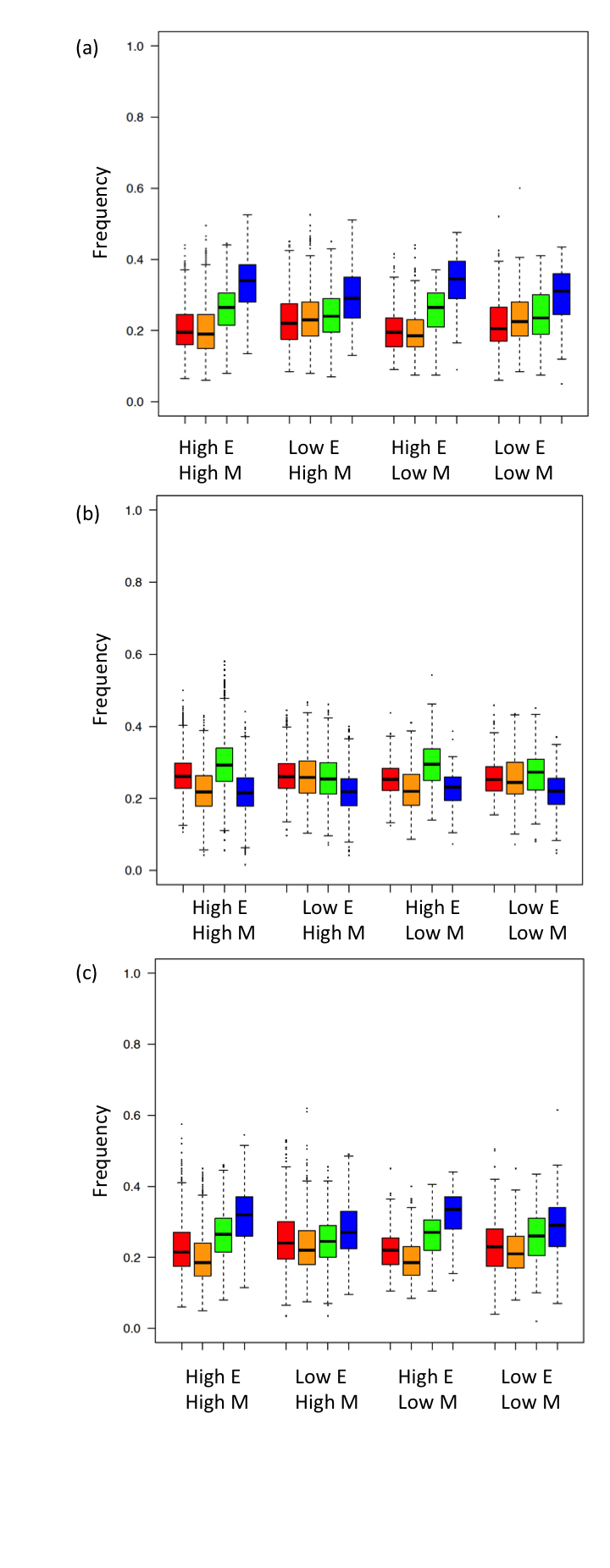


**Figure S16:** Normalized nucleotide frequency at exons and flanking intronic regions (G , C, A , T in red, orange, green and blue, respectively) (a) upstream intron, (b) exon and (c) downstream intron for methylated exons in high expressed genes (HighEhighM), methylated exons in low expressed genes (LowEhighM), hypomethylated in high expressed genes (HighElowM) and hypomethylated exons in low expressed genes (LowElowM).

**Figure S17:** High expressed hypomethylated exons are enriched for overlapping with DNase-I hotspots and predicted enhancer regions in the IMR90 cells. Percentages of overlap with regions determined in (41) as (a) DNase-I hotspots or (b) enhancer regions in IMR90 are shown for hypomethylated exons (HM) and methylated exons (M) for both the low expressed and high expressed exon sets.


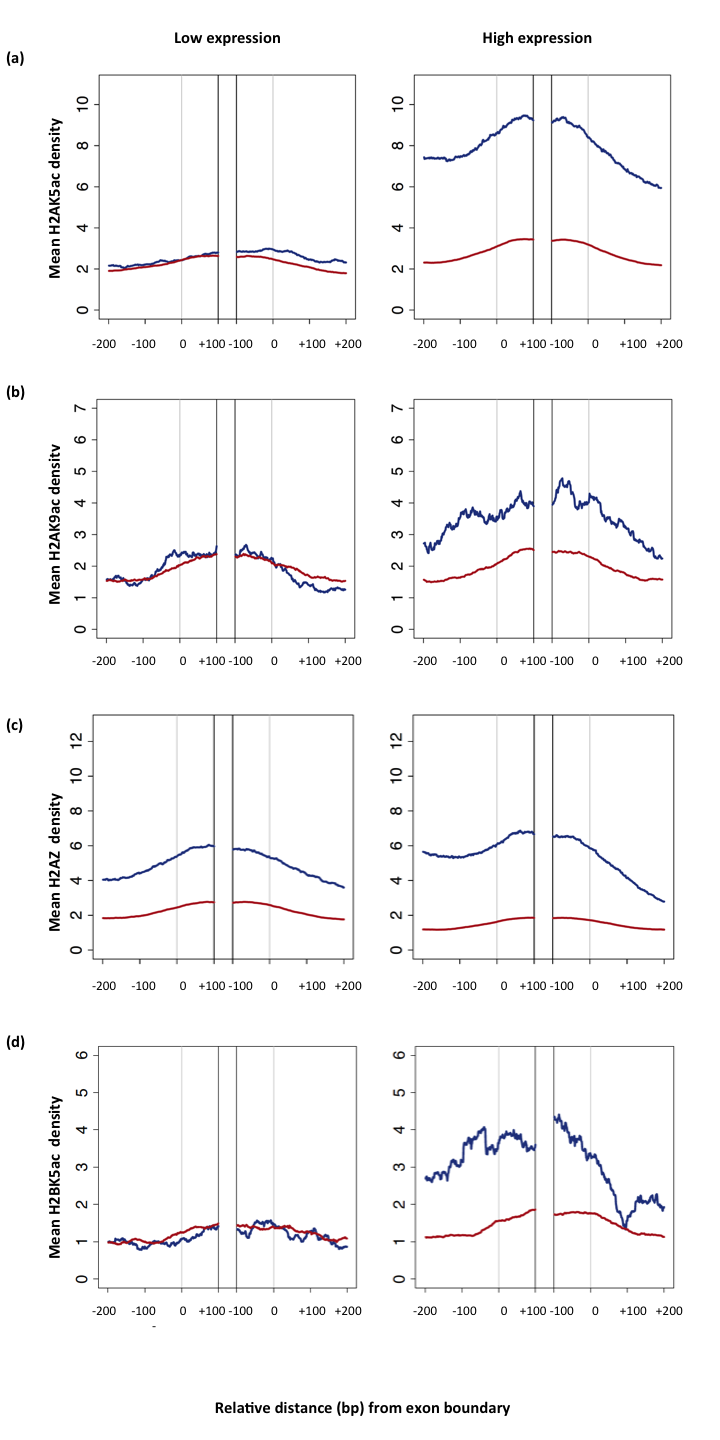


**
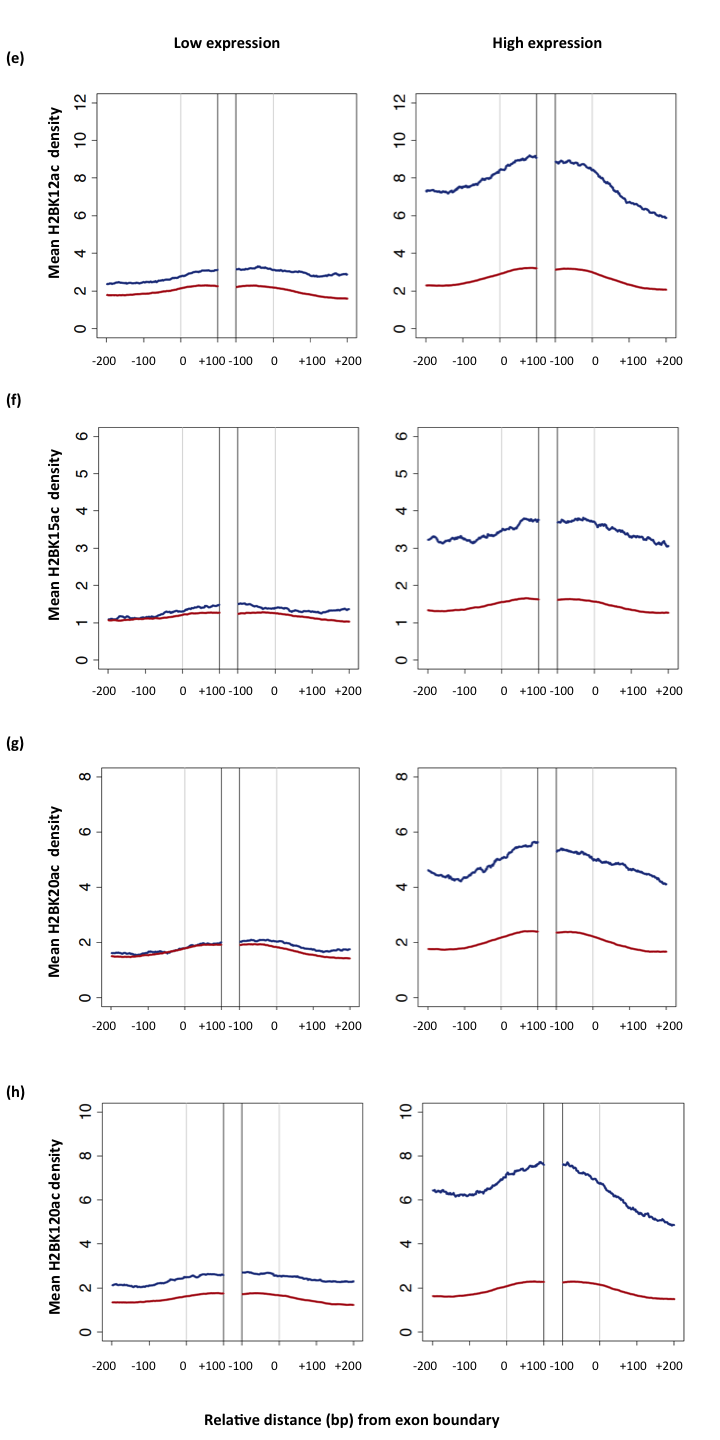
**

**
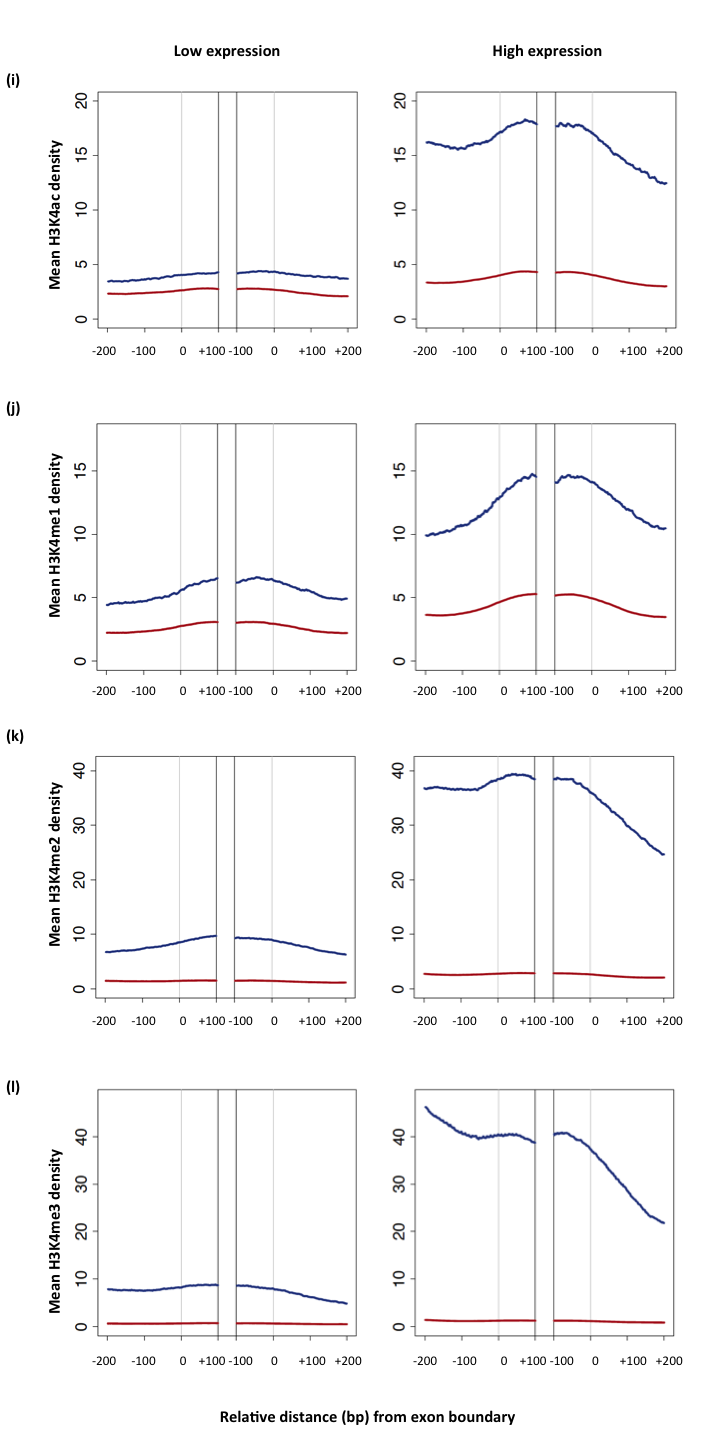
**

**
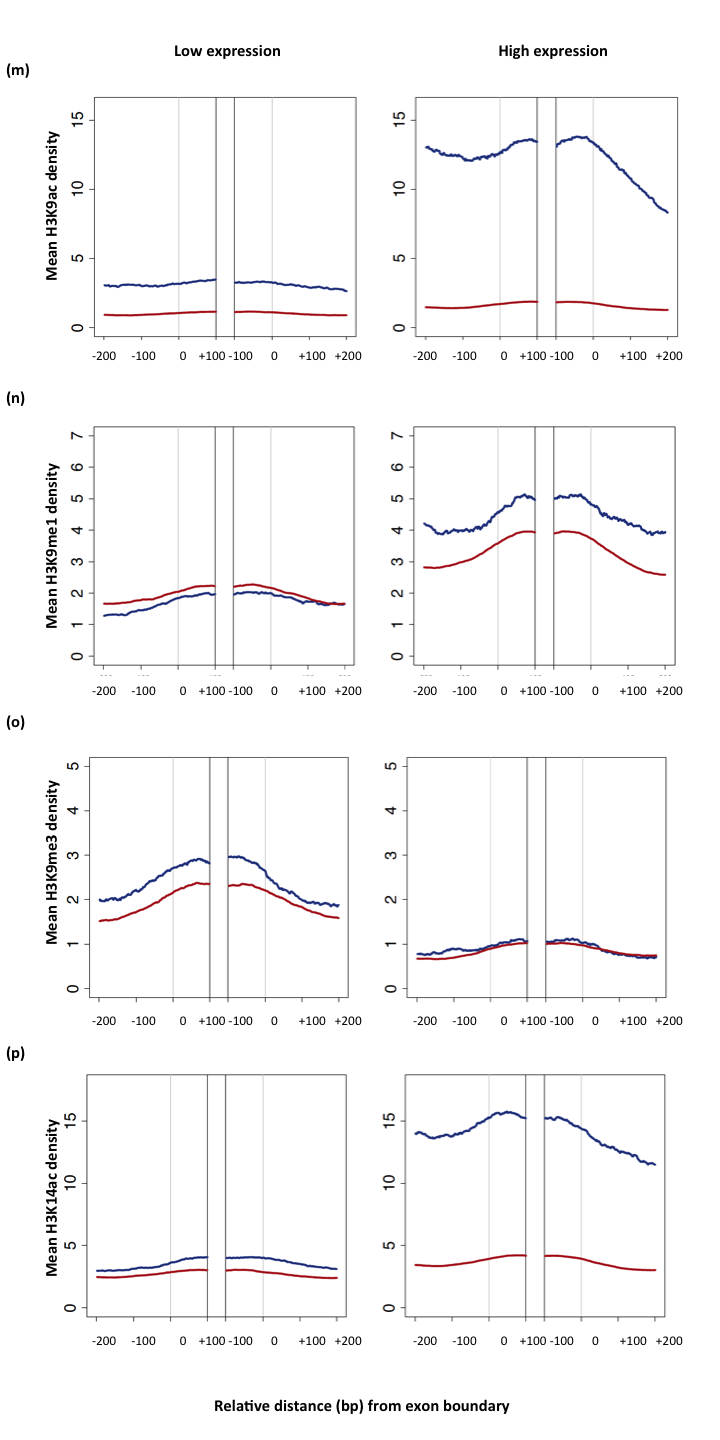
**

**
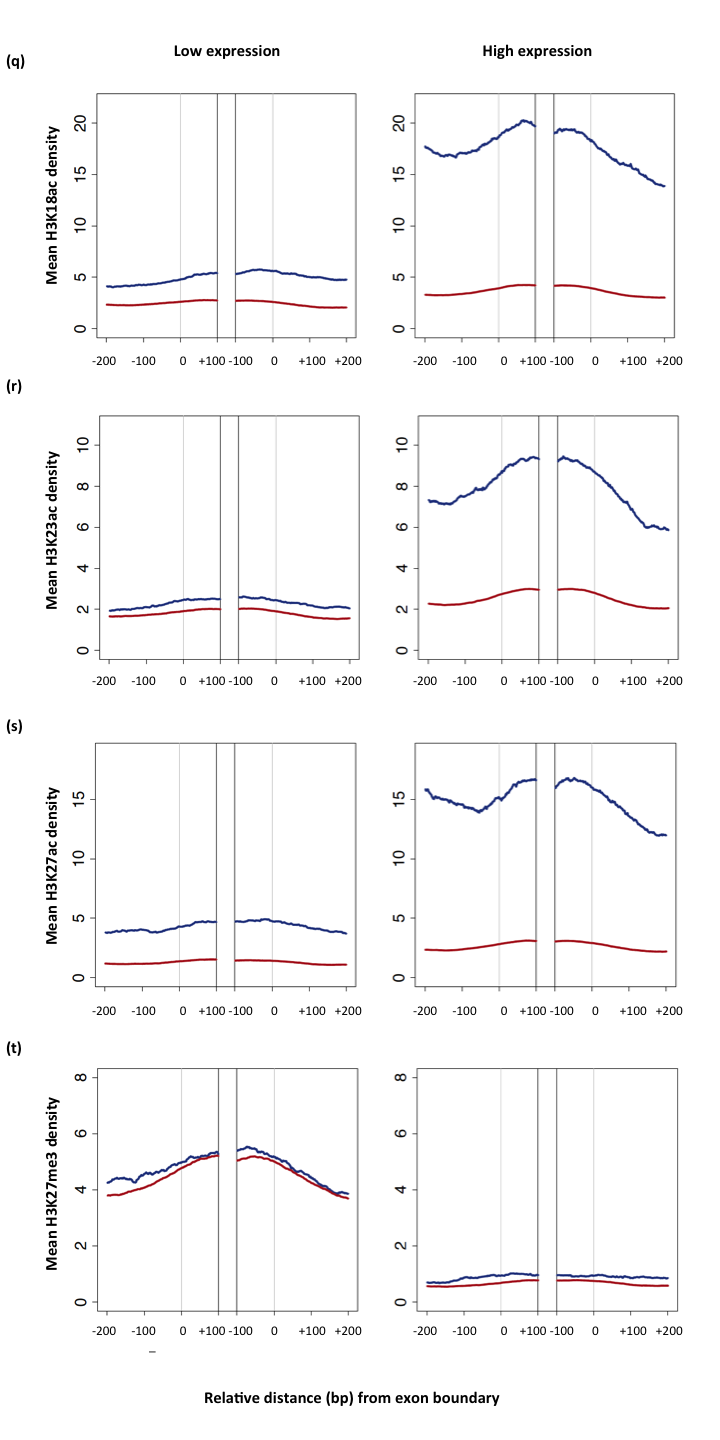
**

**
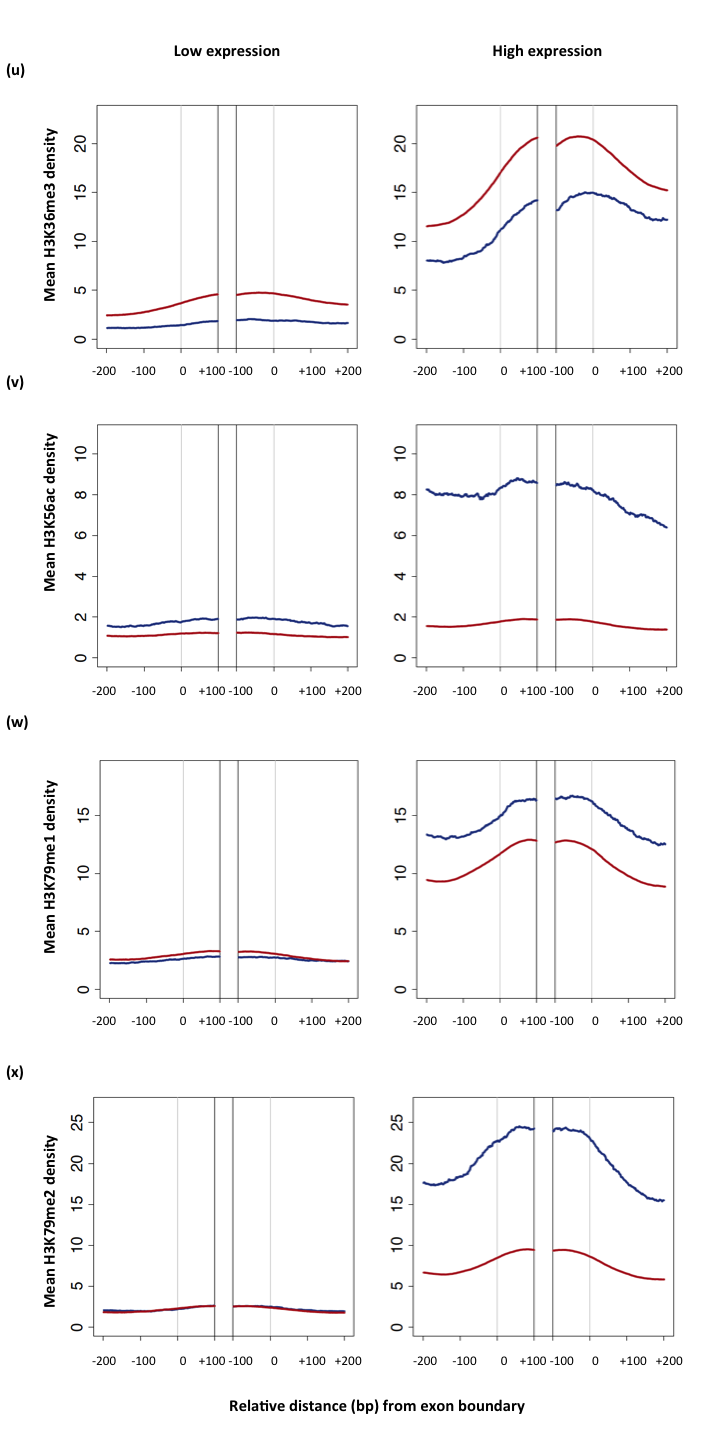
**

**
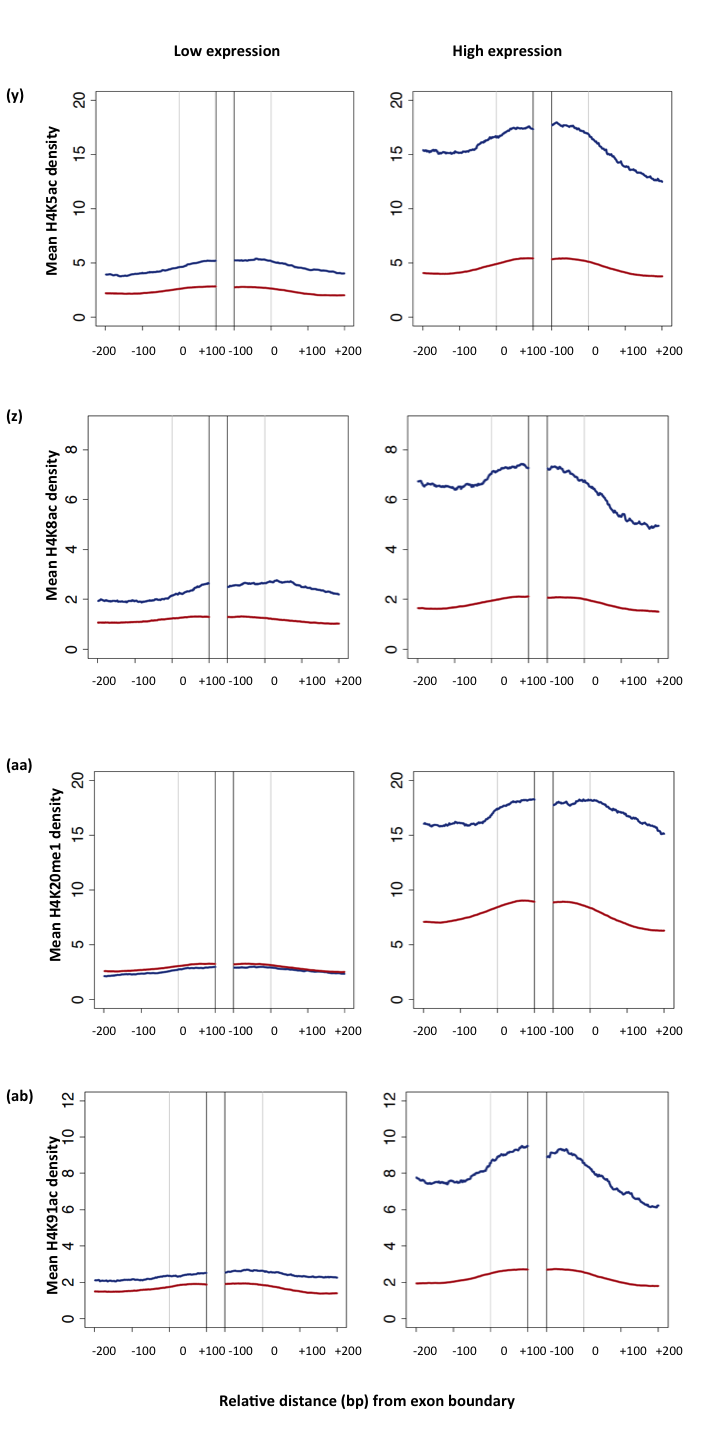
**

**Figure S18:** Histone modifications at different expression rates: The bottom 20-th percentile for expression (left) and the top 20-th percentile for expression (right), for hypomethylated (blue) and methylated (red) exon sets. (a) H2AK5ac, (b) H2AK9ac, (c) H2AZ, (d) H2BK5ac, (e) H2BK12ac, (f) H2BK15ac,

(g) H2BK20ac, (h) H2BK120ac, (i) H3K4ac, (j) H3K4me1, (k) H3K4me2,

(l) H3K4me3, (m) H3K9ac, (n) H3K9me1, (o) H3K9me3, (p) H3K14ac, (q) H3K18ac, (r) H3K23ac, (s) H3K27ac, (t) H3K27me3, (u) H3K36me3, (v) H3K56ac,

(w) H3K79me1, (x) H3K79me2, (y) H4K5ac, (z) H4K8ac, (aa) H4K20me1,

(ab) H4K91ac.

**Figure S19:** Mean H3 density at the bottom 20-th percentile of expression (left) and the top 20-th percentile of expression (right), for all (grey) the hypomethylated (blue) and methylated (red) exon sets.

**Figure S20:** An integrated analysis of the histone modification densities at hypomethylated (in blue) and methylated (in red) exons of different expression rates, restricted to exons that are at least 2Kb away from any TSS.


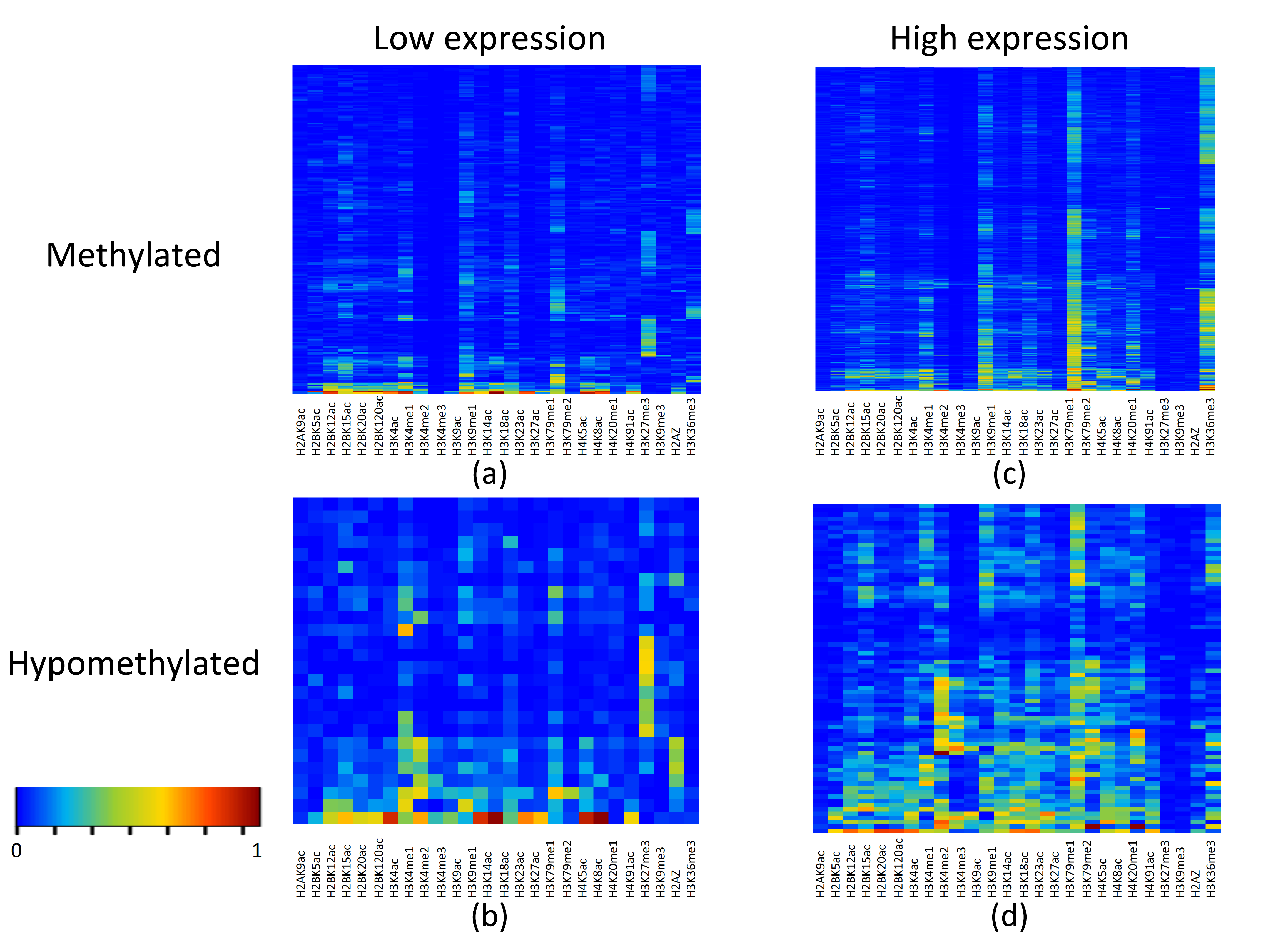


**Figure S21:** Normalized average densities of 28 histone modifications at exons with different expression and methylation rates for bottom 20th percentile for expression (left): (a) methylated exons, (b) hypomethylated exons and top 20-th percentile for expression (right): (c) methylated exons, (d) hypomethylated exons.
