# Supplementary material for: A diverse epigenetic landscape at human exons with implication for expression
Source: Nucleic Acids Res. 2015 Mar 12;43(7):3498–508. doi: 10.1093/nar/gkv153 (PMC4402514; doi:10.1093/nar/gkv153)
Supplement: SUPPLEMENTARY DATA [file supp_gkv153_nar-02745-a-2014-File008.docx]

**Table S1:** Statistics of the analyses at exons in IMR90.

|  | Median methylation rate (SD) | | | Median GC rate (SD) | | | Count of normalized set |
| --- | --- | --- | --- | --- | --- | --- | --- |
|  | Up-stream | Exon | Down-stream | Up-stream | Exon | Down-stream |  |
| Intragenic Exons (IE) | 0.91 (0.23) | 0.93 (0.19) | 0.91 (0.22) | 0.38 (0.11) | 0.48 (0.06) | 0.40 (0.11) | 14487 |
| High Expression IE | 0.93 (0.20) | 0.95 (0.16) | 0.94 (0.18) | 0.38 (0.11) | 0.48 (0.08) | 0.40 (0.11) | 2897 |
| Low Expression IE | 0.74 (0.26) | 0.83 (0.23) | 0.77 (0.26) | 0.46 (0.11) | 0.52 (0.08) | 0.46 (0.11) | 1657 |
| Cassette Exons of Highly Expressed Genes (CE) | 0.91 (0.25) | 0.93 (0.23) | 0.92 (0.22) | 0.43 (0.10) | 0.43 (0.12) | 0.43 (0.10) | 377 |
| High Inclusion CE | 0.92 (0.26) | 0.94 (0.22) | 0.92 (0.22) | 0.44 (0.12) | 0.46 (0.11) | 0.46 (0.13) | 88 |
| Low Inclusion CE | 0.90 (0.24) | 0.92 (0.21) | 0.91 (0.22) | 0.43 (0.10) | 0.42 (0.12) | 0.44 (0.10) | 157 |
| Methylated  Intragenic Exons (M-IE) | 0.92 (0.19) | 0.93 (0.11) | 0.92 (0.18) | 0.38 (0.11) | 0.48 (0.06) | 0.40 (0.11) | 13506 |
| High Expression M-IE | 0.94 (0.16) | 0.95 (0.08) | 0.94 (0.15) | 0.41 (0.11) | 0.50 (0.06) | 0.40 (0.09) | 2779 |
| Low Expression M-IE | 0.77 (0.23) | 0.86 (0.13) | 0.79 (0.23) | 0.46 (0.11) | 0.52 (0.08) | 0.48 (0.11) | 1450 |
| Hypomethylated  Intragenic Exons (H-IE) | 0.39 (0.31) | 0.29 (0.16) | 0.46 (0.31) | 0.38 (0.11) | 0.47 (0.08) | 0.40 (0.11) | 947 |
| High Expression H-IE | 0.34 (0.35) | 0.26 (0.17) | 0.47 (0.33) | 0.39 (0.11) | 0.46 (0.09) | 0.39 (0.15) | 117 |
| Low Expression H-IE | 0.36 (0.28) | 0.30 (0.16) | 0.45 (0.30) | 0.46 (0.12) | 0.52 (0.09) | 0.46 (0.11) | 202 |

*SD – Standard deviation.

|  | Median methylation rate (SD) | | | Median GC rate (SD) | | | Count of normalized set |
| --- | --- | --- | --- | --- | --- | --- | --- |
|  | Up-stream | Exon | Down-stream | Up-stream | Exon | Down-stream |  |
| Intragenic Exons (IE) | 0.91 (0.20) | 0.93 (0.17) | 0.91 (0.19) | 0.44 (0.11) | 0.51 (0.09) | 0.46 (0.11) | 14721 |
| High Expression IE | 0.93 (0.20) | 0.95 (0.17) | 0.93 (0.18) | 0.37 (0.09) | 0.46 (0.07) | 0.39 (0.10) | 2944 |
| Low Expression IE | 0.87 (0.20) | 0.90 (0.18) | 0.86 (0.20) | 0.49 (0.11) | 0.54 (0.08) | 0.50 (0.11) | 1215 |
| Cassette Exons of Highly Expressed Genes (CE) | 0.90 (0.28) | 0.92 (0.26) | 0.91 (0.25) | 0.41 (0.10) | 0.48 (0.09) | 0.43 (0.10) | 259 |
| High Inclusion CE | 0.92 (0.24) | 0.93 (0.22) | 0.91 (0.21) | 0.35 (0.08) | 0.45 (0.07) | 0.42 (0.07) | 46 |
| Low Inclusion CE | 0.89 (0.30) | 0.92 (0.26) | 0.9 (0.24) | 0.47 (0.10) | 0.54 (0.09) | 0.47 (0.11) | 101 |
| Methylated  Intragenic Exons (M-IE) | 0.91 (0.16) | 0.94 (0.09) | 0.92 (0.15) | 0.44 (0.11) | 0.51 (0.08) | 0.46 (0.11) | 13999 |
| High Expression M-IE | 0.93 (0.16) | 0.95 (0.09) | 0.93 (0.15) | 0.37 (0.09) | 0.46 (0.07) | 0.39 (0.09) | 2821 |
| Low Expression M-IE | 0.88 (0.16) | 0.91 (0.10) | 0.88 (0.16) | 0.48 (0.11) | 0.55 (0.08) | 0.50 (0.10) | 1145 |
| Hypomethylated  Intragenic Exons (H-IE) | 0.40 (0.33) | 0.27 (0.16) | 0.40 (0.33) | 0.50 (0.12) | 0.53 (0.10) | 0.50 (0.12) | 684 |
| High Expression H-IE | 0.33 (0.35) | 0.26 (0.17) | 0.44 (0.31) | 0.41 (0.11) | 0.47 (0.09) | 0.43 (0.12) | 121 |
| Low Expression H-IE | 0.42 (0.34) | 0.27 (0.17) | 0.33 (0.30) | 0.52 (0.13) | 0.55 (0.10) | 0.52 (0.13) | 65 |

**Table S2:** Statistics of the analyses at exons in B cells.

*SD – Standard deviation.
